# An Efficient Method for Isolation of Plasmid DNA for Transfection of Mammalian Cell Cultures

**DOI:** 10.3390/mps3040069

**Published:** 2020-10-14

**Authors:** Daniel V. Kachkin, Julia I. Khorolskaya, Julia S. Ivanova, Aleksandr A. Rubel

**Affiliations:** 1 Laboratory of Amyloid Biology, St. Petersburg State University, 199034 St. Petersburg, Russia; 2 Department of Genetics and Biotechnology, St. Petersburg State University, 199034 St. Petersburg, Russia; 3 Institute of Cytology Russian Academy of Science, 194064 St. Petersburg, Russia; khorolskaya@incras.ru (J.I.K.); ju.s.ivanova@yandex.ru (J.S.I.)

**Keywords:** plasmid, mammalian cell transfection, lentiviral transduction, lentivirus, HEK293T, *E. coli*

## Abstract

In this article, we present several protocols that describe the steps from cloning and obtaining a large amount of pure plasmid DNA to generation of lentiviruses based on these constructs. The protocols have been worked out on human cell culture HEK293T but can be adapted for other cell cultures. This protocol was designed to be simple to execute and cheap since it requires only materials and consumables widely available in molecular laboratories, such as salts, alcohols, etc., and no complicated laboratory equipment. These protocols are highly effective and can be performed in any standard molecular biology laboratory.

## 1. Introduction

Transfection is a procedure that introduces DNA and RNA into eukaryotic cells to produce genetically modified cells. Transfection is a powerful analytical tool for studying gene function and their regulation by enhancing or inhibiting specific gene expression in cells. It is also important for studying proteins or producing recombinant proteins in mammalian cells [1]. Transfection can be used in gene therapy, for production of recombinant proteins for therapeutic purposes, small interference RNA knockdown procedures [2] and is an essential step for lentivirus production [3].

There are various approaches for mammalian cell transfection. However, one of the key factors for successful transfection remains the quality and the quantity of the transfected material (DNA, RNA). To transfect human cells, a researcher needs a large amount of high-quality DNA. DNA midi- or maxiprep kits are often expensive and the amount of DNA isolated with their help is often insufficient.

In this study, we developed a new protocol suitable to achieve a high yield of plasmid DNA of high purity, acceptable for the transfection of mammalian cell cultures. Our method can also significantly reduce the cost of DNA isolation, since it provides a high yield of extracted plasmid without an implementation of commercial kits.

For this method, we used the *E. coli* bacterial strain *STBL3* (ThermoFisher Scientific, Walthon, MA, USA). This strain was designed especially for cloning the direct repeats found in lentiviral expression vectors and it gives a higher yield of extracted DNA. However, the introduced protocol is probably compatible with any *E. coli* strain used for the cloning.

The DNA purity is sufficient for basic transfection methods using cationic polymers, such as PEI (polyethylenimine) or TurboFect (ThermoFisher, Walthon, MA, USA). Transfection using PEI has several advantages, like its cheapness and suitability for various cell cultures [4].

Lentiviral technology came to widespread use in molecular and cell biology for stable gene expression, gene silencing, generating transgene animals, induction of pluripotent stem cells, stem cell modification and lineage tracking, immunization and in vivo imaging [5]. Lentiviral technology is based on the co-transfection of human cell line HEK293T with packaging, envelope and transfer plasmids (containing the gene of interest). This is resulted in the assembly of the lentivirus, which, upon transduction, became a powerful tool for the expression of exogenous genes into various types of cells both in vitro and in vivo. Today, this method is the most effective way to obtain stable cell lines producing the studied genetic products [6].

These protocols can be easily reproduced in almost any lab, they have a good efficiency and may be necessary for the wide range of studies in cell and molecular biology.

In this article, we offer well-reproducible, high-performance protocols that are guided and adapted to work with mammalian cell cultures. This protocol of plasmid DNA extraction is also applicable to other techniques where a large amount of plasmid DNA is required.

## 2. Experimental Design

**Figure 1:**
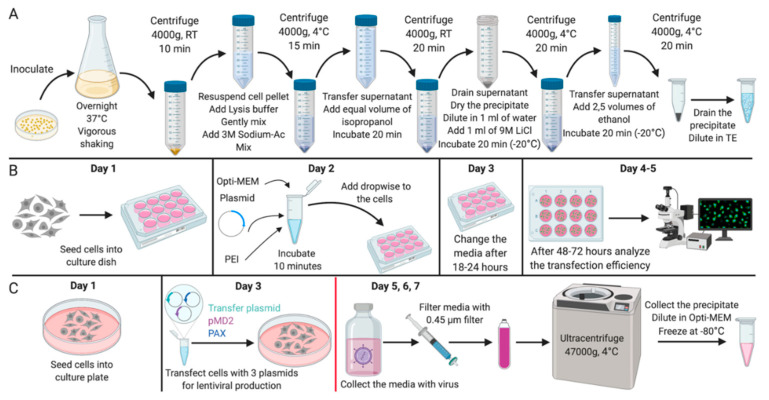
Experimental design. Scheme of (**A**) the experiment for the isolation of plasmid DNA; (**B**) the experiment for the mammalian cells transfection; (**C**) the experiment for the lentiviral vector production. All stages after the change of media on Day 4 should be carried out with the proper biosafety containment recommended for research with lentiviral vectors. Created with BioRender.com.

In this article, we propose an optimized protocol for the isolation of plasmid DNA (Figure 1) from bacterial cultures without the use of commercial kits for its subsequent application in genetic modifications of mammalian cell cultures. The advantages of this method are as follows: a high efficiency (concentration of plasmid DNA at the output is 3–8 mg/mL), availability, sufficient purity of the obtained material, as well as cheapness along with relatively low time costs. In addition, the protocol contains a description of the methods for efficient transfection and assembly of lentiviral particles based on the obtained plasmid DNA, which have been successfully tested for immortalized and primary mammalian cell cultures.

We recommend using plasmids containing fluorescent labels, as this will provide the best control over the experiment, calculation of transfection/transduction efficiency and viral assembly.

The plasmids obtained for the study were transformed into *E. coli* according to the Inoue standard protocol [7]. We used the plasmids pLenti-CMV-EGFP Hygro (656-4) [8], pMD2 (gift from Didier Trono (Addgene plasmid # 12259)) and PAX2 (gift from Didier Trono (Addgene plasmid # 12260)). The protocol is based on the Birnboim and Doly DNA extraction protocols with modifications [9,10].

This protocol lists the plasmids and strain used in this study, but the DNA extraction protocol is suitable for all strains and plasmids. It is suitable for all researchers working with cell cultures due to its simplicity and it does not require complicated equipment or expensive reagents.

### 2.1. Materials

- Tris base (ThermoFisher Scientific, USA; Cat. no.:15504020);
- EDTA Disodium Salt 2-hydrate (PanReac AppliChem, USA; Cat. no.:141669);
- Yeast Extract w/o salts (Helicon, Russia; Cat. no.: H-0601MG-0.5);
- D-Glucose monohydrate (Helicon, Russia; Cat. no.: Roquette-361103-0.5);
- Tryptone (VWR Life Science AMRESCO, USA; Cat. no.: 97063-388);
- Agar, European type (PanReac AppliChem, USA; Cat. no.: 402302.1210-1);
- Sodium Cloride (Helicon, Russia; Cat. no.: H-1418-1.0);
- Lithium Chloride (Sigma-Aldrich, USA; Cat. no: L4408);
- Sodium Acetate (PanReac AppliChem, USA; Cat. no.: 131633);
- Sodium hydroxide (PanReac AppliChem, USA; Cat. no.: 211687);
- Sodium Dodecyl Sulfate (PanReac AppliChem, USA; Cat. no.: 142363);
- RNAse A (Sigma-Aldrich, USA; Cat. no: R6148);
- Antibiotic for plasmid selection (we used Ampicillin (Sigma-Aldrich, USA; Cat. no.: 10835242001));
- Ethanol;
- Isopropanol;
- Dulbecco’s Modified Eagle Medium (DMEM; Gibco, USA; Cat. no.: 41965039);
- Opti-MEM Reduced-Serum Medium (Gibco, USA; Cat. no.: 51985026);
- Defined Fetal Bovine Serum, (FBS; HyClone, USA; Cat. no.: SH30070.03);
- L-glutamine (Gibco, USA; Cat. no.:25030024);
- Penicillin-streptomycin (Gibco, USA; Cat. no.:15070063);
- Polyethylenimine (PEI) HCl MAX, MW 40000 (Polysciences, USA; Cat. no.: 24765-1);
- Hexadimethrine bromide (Polybrene) (Sigma-Aldrich, USA; Cat. no.: 107689);
- Bacterial strain *STBL3* (ThermoFisher Scientific, USA);
- Human cell line HEK293T (Cells were obtained from the Russian Cell Culture Collection (Institute of Cytology, St. Petersburg, Russia));
- pMD2—envelope plasmid (gift from Didier Trono, addgene plasmid # 12259);
- PAX2—packaging plasmid (gift from Didier Trono, addgene plasmid # 12260);
- pLenti-CMV-GFP Hygro (656-4)—transfer lentiviral plasmid [8].

### 2.2. Equipment

- Tissue culture-treated plates (TPP Techno Plastic Products AG Schaffhausen, Trasadingen, Switzerland);
- 15 mL Falcon Conical Centrifuge Tubes (Corning, Corning, USA; Cat. no.: 352095);
- 50 mL Conical Centrifuge Tubes (Corning, Corning, NY, USA; Cat. no: 352070);
- Syringe 0.45-μm filter (Jet Bio-Filtration, Guangzhou, China; Cat. no.: FPE404030);
- Polycarbonate centrifuge bottles (Beckman Coulter, Brea, CA, USA; Cat. no: 363420);
- New Brunswick Galaxy 170R CO_2_ incubator (Eppendorf, Hamburg, Germany);
- Class II, Type A2 Biological Safety Cabinets (Lamsystems, Miass, Russia);
- ZOE Fluorescent cell imager (Bio-Rad, Hercules, CA, USA);
- Inverted microscope Eclipse TS100 (Nikon, Tokyo, Japan);
- Vortex Genius 3 (IKA, Staufen, Germany);
- Shaker incubator (Biosan, Riga, Latvia);
- Petri dishes for microbiology (Helicon, Moscow, Russia);
- Centrifuge with cooling Eppendorf 5810 R (Eppendorf, Hamburg, Germany);
- Avanti J-E Centrifuge (Beckman Coulter, Brea, CA, USA; Cat. no: 369005);
- CytoFLEX Flow Cytometer (Beckman Coulter, Brea, CA, USA);
- Spectrophotometer Nanodrop TB-1000, ThermoFisher Scientific, Walthon, MA, USA).

## 3. Procedure

### 3.1. Plasmid DNA Isolation from E. coli Bacteria—Time for Completion: 16–18 h

1. Inoculate a single colony transformed with the desired plasmid from the plate with LB (+ antibiotic for selection) into 50 mL of liquid medium (LB or TBR) with the appropriate antibiotic added.
  Note: The flask for growing the bacteria should be at least 4–5 times larger than the volume of the medium in which the bacteria grow. This is necessary for a better aeration of the culture.
2. Grow the cells with vigorous shaking overnight at 37 °C.
3. Transfer the cells to centrifuge tubes (V = 50 mL) and centrifuge at 4000× *g* for 10 min at room temperature.
4. Carefully drain the supernatant and resuspend the cell pellet in 5 mL of sterile water or TEG with the addition of RNAse (3–5 μL/mL). Add TEG (Tris-EDTA-Glucose buffer) at sterile conditions, to avoid contamination.
5. Add 10 mL of a lysis solution (0.2N NaOH; 1% SDS) to the cell suspension. Gently mix by inverting the tube until all cells are lysed. It is important to mix the tube gently by inverting to avoid contamination by genomic DNA.
6. Add 7.5 mL of 3M sodium acetate (pH = 5.0) and mix thoroughly. Incubate the sample for 10 min at 4 °C.
  Note: If the precipitate does not settle well or flocs remain in the solution after the centrifugation, then the volume of the sodium acetate solution may be increased up to 10 mL or its pH is brought to 4.8.
7. Centrifuge the sample for 15 min at 4000× *g* at 4 °C.
8. Transfer the supernatant that contains the plasmid DNA to a new tube and add an equal volume of isopropanol. Thoroughly mix and incubate the sample for 15 min at room temperature. It is important to use high quality isopropanol as this directly affects the purity of the DNA.
9. Centrifuge for 20 min at 4000× *g* at room temperature.
10. Drain the supernatant and dry the precipitate. Dissolve the precipitate in 1 mL of sterile water.
11. Add 1 mL of 9M LiCl to the sample, mix thoroughly and incubate 20 min at −20 °C.
12. Centrifuge for 20 min at 4000× *g* at −4 °C.
13. Carefully transfer the supernatant to a new centrifuge tube (V = 15 mL). Discard the precipitate (it contains RNA and protein residues).
14. Add 5 mL of 96% ethanol to the supernatant. Vortex thoroughly.
15. Incubate for 1 h at −20 °C.

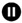
**PAUSE STEP** It is possible to pause the protocol here and leave samples at −20 °C overnight.

16. Centrifuge at 4000× *g* for 20 min at 4 °C.
17. Discard the supernatant and thoroughly dry the precipitate.
18. Dissolve the plasmid DNA in 500 μL of sterile water or TE buffer.

### 3.2. Transfection of Human Cell Culture HEK293T Using PEI—Time for Completion: 3–5 Days

1. One day before the transfection, seed HEK293T cells into a multi-well culture plate or a tissue culture dish with respect to the required number of cells per 1 cm^2^ (the approximate number of cells for different culture dishes is shown in Table 1). Cultivate the cells overnight in DMEM supplemented with 10% FBS, 1% L-glutamine, and 1% penicillin/streptomycin.
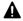
**CRITICAL STEP** It is crucial to check the cells for mycoplasma contamination; the presence of mycoplasma may significantly reduce the efficiency of the transfection or transduction.
2. Next day right before transfection prepare the DNA and PEI solution (transfection mix). The volume of transfection mix is 10% of the total volume of the culture medium (recommended by the manufacturer of culture dishes). Add the required amount of plasmid DNA (see Table 1) to the Opti-MEM and mix well on a vortex. Then, dropwise add PEI (1 mg/mL) to the DNA solution while vortexing.
  Note: We recommend using plasmid DNA in a final concentration 1–2 mg/mL. The amount of PEI may vary according to the amount of DNA. Avoid using concentrations higher than 5 μL/mL due to its high toxicity.
3. Incubate the transfection mix at room temperature for 10–15 min.
4. Remove the medium from the cells and add the required amount of fresh DMEM supplemented with 10% FBS, 1% L-glutamine, and 1% penicillin/streptomycin.
  Note: The presence/absence of an antibiotic in the medium does not affect the efficiency of transfection. It is possible to use Opti-MEM instead of DMEM.
5. Add the transfection mix dropwise to the cells with a fresh culture medium and mix by gently swirling the culture dish.
6. Incubate the cells with the transfection mix in a tissue culture incubator at 37 °C, 5% CO_2_ for 18–24 h.
7. After the incubation, aspirate the medium with the transfection mix from the cells and add the required amount of DMEM supplemented with 10% FBS, 1% L-glutamine, and 1% penicillin/streptomycin.
8. After 48–72 h of transfection, the effectiveness of the transfection can be evaluated. Note: For plasmids with fluorescent protein the effectiveness of the transfection can be evaluated by flow cytometry or fluorescent microscope. For other proteins use antibody or other staining.

### 3.3. Lentiviral Vector Production—Time for Completion: 5–7 Days

#### 3.3.1. Preparing Cells and Transfection

1. Seed 1 × 10^6^ HEK293T cells into a 100 mm culture plate three days before the planned transfection with lentiviral vector plasmids. On the day of transfection, cells should cover about 70% of the plate surface.
  Note: Take four 100 mm culture plates for production of a sufficient amount of vector for further research. For small-scale purposes, one well of a 6-well plate may be enough.
  **OPTIONAL STEP** We recommend to seed cells on Friday and make the transfection on Monday after the weekend so the whole process will be ended at the end of the working week.
  **OPTIONAL STEP** We recommend to coat the surface of culture plates with gelatin (ATCC, USA) or collagen (Sigma-Aldrich, USA) for better adhesion of cells during experiments.
2. Transfect cells with three plasmids necessary for the virus production: pLenti-CMV-GFP Hygro (656-4) [8], PAX2 and pMD2. Right before transfection, prepare the DNA and PEI solutions. The volume of transfection mix is 10% of the total volume of the culture medium. Add the required amount of plasmid DNA (see Table 2) to the Opti-MEM and mix well on a vortex. Then, dropwise add PEI (1 mg/mL) to the DNA solution while vortexing.
3. Incubate the transfection mix at room temperature for 10–15 min.
4. Change the media containing the transfection mix to fresh DMEM supplemented with 10% FBS, 1% L-glutamine, and 1% penicillin/streptomycin.
5. Add the transfection mix dropwise to the cells with a fresh culture medium and mix by gently swirling the culture dish.
6. Incubate the cells with the transfection mix in a tissue culture incubator at 37 °C, 5% CO_2_ for 18–24 h.
7. The next day after transfection, change the medium to fresh DMEM supplemented with 10% FBS, 1% L-glutamine, and 1% penicillin/streptomycin.
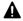
**CRITICAL STEP** This and all following steps must be done with the proper biosafety containment recommended for research with lentiviral vectors [11].

#### 3.3.2. Harvesting the Lentiviral Vectors

8. The next three days, collect daily the medium containing the virus in sterile 50-mL centrifuge tubes. Store the medium at +4 °C for up to one week.
9. On the fifth day after transfection, the collected medium must be filtered with a filter with a 0.45 μm filter to get rid of cell debris in the medium.
  **OPTIONAL STEP** For an easier filtration, it is possible to preliminarily centrifuge the collected medium (3 min at 500× *g*) to precipitate the main cell debris.

#### 3.3.3. Ultracentrifugation

10. Transfer the filtered virus-containing medium to sterile polycarbonate centrifuge bottles suitable for ultracentrifugation and balance them carefully using suitable weights (to 3 decimal points).
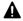
**CRITICAL STEP** the 50 mL centrifuge tubes must be strong enough to withstand a centrifugation speed of 50,000× *g*.
11. Centrifuge the tubes for 2 h at 47,000× *g*, at +4 °C.
  Note: After the centrifugation, at the bottom of the tube will be a barely noticeable whitish precipitate.
12. Take away the supernatant without touching the precipitate.
13. Thoroughly dissolve the precipitate in 150 μL of Opti-MEM per 50 mL of harvested vector-containing medium by pipetting.
14. Leave them for 1 h at room temperature for better dissolution.
15. Centrifuge samples at low speed (2000× *g*, 3 min) to collect the media with the virus particles.
16. Aliquot samples into 20–50 μL portions and freeze them at −80 °C. In this state, the samples can be stored for a very long time.
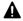
**CRITICAL STEP** We strongly recommend neither to repeat the freezing/thawing cycles for the virus nor to store it for a long time at +4 °C and at room temperature, since its transduction efficiency can be significantly reduced.

#### 3.3.4. Viral Titer Calculation

17. Seed 5 × 10^4^ HEK293T cells into a 24-well plate the day before transduction. Cultivate the cells overnight in DMEM supplemented with 10% FBS, 1% L-glutamine, and 1% penicillin/streptomycin.
18. The next day, prepare the 10-fold serial dilution of your lentivirus in 500 μL of Opti-MEM.
  **OPTIONAL STEP** To increase the efficiency of the transduction, use hexadimethrine bromide (polybrene) (Sigma-Aldrich, USA) in concentration 5 μg/mL. This step is essential for the primary lines.
19. Change the culture medium in the culture plate to the Opti-MEM medium with different dilutions of the virus (see Point 18) to infect the cells.
20. Incubate the cells in a tissue culture incubator at 37 °C, 5% CO_2_ for 18–24 h.
21. The next day after transduction, change the medium to fresh DMEM supplemented with 10% FBS, 1% L-glutamine, and 1% penicillin/streptomycin.
22. Two days after transduction, perform a flow cytometry analysis to count the percentage of the infected cells per well. To detect the infected cells, use the fluorescence signal value for viruses containing a fluorescent protein or antibody/other staining for non-fluorescent ones.
23. Choose the well in which the percentage of infected cells do not exceed 20% and calculate the viral titer using the following equation: TU/mL=(P×N×D)/V, where TU/mL is a number of viral particles in 1 mL of viral solution; P—percentage of infected cells (0.01–0.2); N—the number of cells in the well (at the time of seeding); D—the dilution fold of the added vector; and V—the volume (mL) of the added vector.

## 4. Expected Results

During the approbation of the protocol for the isolation of plasmid DNA, we received on average of 5.6 ± 2.1 μg/μL (which is about 56 μg of DNA per 1 mL of overnight culture). We obtained such amounts of plasmid DNA using the bacterial strain *STBL3*.

The purity of the plasmids and the concentration was evaluated using the spectrophotometer NanoDrop (ND-1000). This is a quick method for estimating the amount of extracted DNA, as well as the relative amount of RNA and protein in a sample.

The ratio of absorbance at 260 nm and 230 nm (260/230) is used as a secondary measure of nucleic acid purity. The 260/230 values for pure nucleic acid are often higher than the respective 260/280 values. A good-quality DNA sample should have an absorbance 260/280 ratio of 1.8–2.0 and a 260/230 ratio of 2.0–2.2 [12]. The average absorbance at 260/280 nm for the samples obtained by this method was 1.94 ± 0.09 and the average absorbance at 260/230 nm was 2.23 ± 0.2.

Plasmids extracted with the described method were successfully used for transfection of HEK293T cells and lentivirus production.

Transfection of mammalian cell cultures is a very powerful tool in various biological fields. We used a basic protocol using the cationic polymer PEI. This method is suitable for different purposes and can be done with good efficiency in different stable cell lines. The efficiency of HEK293T transfection on the third day was more than 90% (measured using a CytoFLEX flow cytometer (Beckman Coulter, Brea, CA, USA)).

The protocol for the production of lentiviral vectors is suitable for various lentiviral transfer plasmids with an insert size up to 15 kb [13].

**Figure 2:**
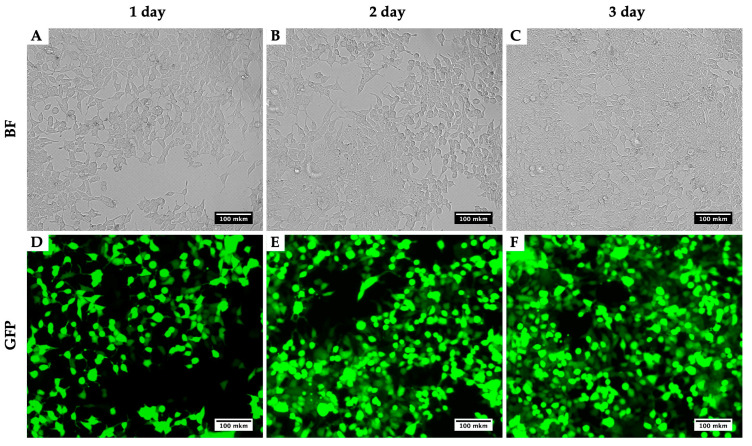
Dynamics of GFP signal accumulation in HEK293T within three days during the LV-CMV-EGFP Hygro (656-4) virus production. BF, brightfield; GFP, detection of green fluorescence. HEK293T cells 24 h (**A**,**D**), 48 h (**B**,**E**) and 72 h (**C**,**F**) after initial transfection with plasmids for LV-CMV-EGFP Hygro (656-4) production.

Using this method for the production of lentiviruses, using the plasmids pMD2 (Addgene plasmid #12259), PAX2 (Addgene plasmid #12260) and pLenti-CMV-GFP Hygro (656-4) [8], we obtained viral titers up to 1.5 × 10^8^ TU/mL. Measurements of the calculation of the efficiency of transduction with lentivirus were carried out on a CytoFLEX flow cytometer (Beckman Coulter, Brea, CA, USA). As an example, we present a photo of HEK293T for three days during the virus production (Figure 2). Figure 2 compares two techniques: the bright field presents a number of cells infected with the virus and their morphological alterations, while GFP depicts the viral assembly. With the three days of growth, an increase in the GFP intensity is noticeable and so too the assembled viral particles.

We describe the obtaining of LV-CMV-EGFP Hygro (656-4) that has a good transduction efficiency with both immortalized (more than 98% efficiency on the third day) and primary mammalian cell lines (60–80% efficiency on the third day, depending on the cell type).

## 5. Reagents Setup

**LB**. 1% tryptone, 0.5% yeast extract, and 1% NaCl. To obtain a solid medium, add agar to a final concentration of 2%. Add deionized water. Shake until the solutes are dissolved and autoclave for 30 min. Store liquid medium at room temperature (or at +4 °C) and add the appropriate antibiotics before use. In the solid medium, add antibiotics after cooling the medium to 45–50 °C and pour into Petri dishes. Store at 4 °C.

**TBR.** Deionized water up to 900 mL, 12 g tryptone, 24 g yeast extract, and 4 mL glycerol. Shake until the solutes are dissolved and autoclave for 30 min. Add 100 mL of sterile solution of 0.17 M KH_2_PO_4_, 0.72 M K_2_HPO_4_ (2.3 g of K_2_HPO_4_ and 12.5 g of KH_2_PO_4_ in 90 mL of H_2_O; after the salts are dissolved, add H_2_O up to 100 mL).

**TEG.** 50 mM Glucose, 25 mM Tris HCl (pH = 8.0), and 10 mM EDTA (pH = 8.0). Add deionized water. Autoclave for 30 min. Can be stored at room temperature.

**Lysis solution.** 0.2 N NaOH (freshly diluted from a 5 N stock) and 1% (w/v) SDS (freshly diluted from a 10% stock). Store solution at room temperature.

**10× TE buffer.** 100 mM Tris-HCl (pH = 8.0), 10 mM EDTA. Sterilize solution by autoclaving for 30 min. Store the buffer at room temperature.

**Polyethylenimine.** For the PEI (1 mg/mL) preparation, see the recommendations written by the manufacturer [14].

## Figures and Tables

**Table 1:** Approximate numbers of cells and reagents for HEK293T transfection.

Tissue Culture Plate	Number of Cells per Well to Seed	Final Medium Volume (mL)	Volume of the Transfection Mix (μL)	Amount of DNA (μg)	Amount of PEI (μL)
48-well plate	40,000	0.5	50	0.5	2.5
24-well plate	80,000	1	100	1	5
12-well plate	150,000	2	200	2	10
6-well plate	300,000	3	300	3	15
60 mm culture dish	1,000,000	5	500	5	25
100 mm culture dish	3,500,000	10	1000	10	50

**Table 2:** Approximate number of cells and reagents for virus production in HEK293T.

Tissue Culture Plate	Number of Cells to Seed Three Days before Transfection	Final Medium Volume (mL)	Volume of Transfection Mix (μL)	Amount of Vector DNA (μg)	Amount of PAX Plasmid (μg)	Amount of pMD2 Plasmid (μg)	Amount of PEI (μL)
6-well plate	100,000	3	300	3.5	2.5	1.3	15
60 mm plate	300,000	6	600	6.5	5	2.5	30
100 mm culture dish	1,000,000	10	1000	11	8	4	50
